# Childhood cancer and residential proximity to power lines

**DOI:** 10.1054/bjoc.2000.1550

**Published:** 2000-11-22

**Authors:** UK Childhood Cancer Study Investigators

**Affiliations:** Writing Committee J Skinner East Anglia region; MP Maslanyj; TJ Mee and SG Allen National Radiological Protection Board; J Simpson Leukaemia Research Fund Data Management Processing Group; E Roman South Midlands region; NE Day East Anglia region A complete list of investigators is given in: The United Kingdom Childhood Cancer Study: objectives, material, and methods. Br J Cancer 2000; 82(5): 10731102. See Appendix for Management Committee, Regional Investigators and Writing Committee

**Keywords:** magnetic fields, childhood cancer, leukaemia, power lines

## Abstract

In the United Kingdom Childhood Cancer Study, a population-based case–control study covering the whole of England, Scotland and Wales, measured power-frequency magnetic fields were not found to be associated with risk for any malignancy. To examine further the risk associated with residential proximity to electricity supply equipment, distances to high-voltage lines, underground cables, substations and distribution circuits were collected for 3380 cases and 3390 controls. Magnetic field exposure from this equipment was calculated using distance, load and other circuit information. There was no evidence that either proximity to electrical installations or the magnetic field levels they produce in the UK is associated with increased risk of childhood leukaemia or any other cancer. Odds ratios of 0.73 (95% CI = 0.42–1.26) for acute lymphoblastic leukaemia, 0.75 (95% CI = 0.45–1.25) for all leukaemias, 1.08 (95% CI = 0.56–2.09) for central nervous system cancers and 0.92 (95% CI = 0.64–1.34) for all malignancies were obtained for residence within 50 m of an overhead line. When individuals with a calculated magnetic field exposure ≥ 0.2 μT were compared to those in a reference category of exposure <0.1 μT, odds ratios of 0.51 (95% CI = 0.11–2.33) for acute lymphoblastic leukaemia, 0.41 (95% CI = 0.09–1.87) for total leukaemia, 0.48 (95% CI =0.06–3.76) for central nervous system cancers and 0.62 (95% CI = 0.24–1.61) for all malignancies were obtained. © 2000 Cancer Research Campaign http://www.bjcancer.com


*British Journal of Cancer* (2000) **83**(11), 1573–1580
© 2000 Cancer Research Campaign
doi: 10.1054/ bjoc.2000.1550, available online at http://www.idealibrary.com on IDEAL®

http://www.bjcancer.com

### Childhood cancer and residential proximity to power lines

**UK Childhood Cancer Study Investigators**

Writing Committee J Skinner, East Anglia region; MP Maslanyj; TJ Mee and SG Allen, National Radiological Protection Board; J Simpson, Leukaemia Research Fund Data Management Processing Group; E Roman, South Midlands region; NE Day, East Anglia region

A complete list of investigators is given in: The United Kingdom Childhood Cancer Study: objectives, material, and methods. *Br J Cancer* 2000; **82**(5): 1073–1102. See Appendix for Management Committee, Regional Investigators and Writing Committee

**Summary** In the United Kingdom Childhood Cancer Study, a population-based case–control study covering the whole of England, Scotland and Wales, measured power-frequency magnetic fields were not found to be associated with risk for any malignancy. To examine further the risk associated with residential proximity to electricity supply equipment, distances to high-voltage lines, underground cables, substations and distribution circuits were collected for 3380 cases and 3390 controls. Magnetic field exposure from this equipment was calculated using distance, load and other circuit information. There was no evidence that either proximity to electrical installations or the magnetic field levels they produce in the UK is associated with increased risk of childhood leukaemia or any other cancer. Odds ratios of 0.73 (95% CI = 0.42–1.26) for acute lymphoblastic leukaemia, 0.75 (95% CI = 0.45–1.25) for all leukaemias, 1.08 (95% CI = 0.56–2.09) for central nervous system cancers and 0.92 (95% CI = 0.64–1.34) for all malignancies were obtained for residence within 50 m of an overhead line. When individuals with a calculated magnetic field exposure ≥ 0.2 μT were compared to those in a reference category of exposure <0.1 μT, odds ratios of 0.51 (95% CI = 0.11–2.33) for acute lymphoblastic leukaemia, 0.41 (95% CI = 0.09–1.87) for total leukaemia, 0.48 (95% CI =0.06–3.76) for central nervous system cancers and 0.62 (95% CI = 0.24–1.61) for all malignancies were obtained. © 2000 Cancer Research Campaign http://www.bjcancer.com

**Keywords:** magnetic fields; childhood cancer; leukaemia; power lines

The UK Childhood Cancer Study (UKCCS) was a population-based case–control study covering England, Scotland and Wales (UK Childhood Cancer Study Investigators, 2000). The study found no association between measured power-frequency magnetic field exposure and risk for any malignancy (UK Childhood Cancer Study Investigators, 1999). Details of high-voltage lines, underground cables, substations and distribution circuits were collected from electricity companies for the homes of most cases and one control per case. Here, we use this information to investigate whether proximity to electricity supply equipment or magnetic field exposure calculated from distance, load and other circuit information is associated with an increased risk of childhood cancer. We focus on the year prior to diagnosis, to investigate whether these exposures might play a role in the promotion or progression of childhood cancers. An examination of electric fields, both measured and calculated, will be published separately.

### MATERIALS AND METHODS

### Study participants

The study population was a subset of participants in the UKCCS, a wide-ranging investigation into several possible causes of childhood cancer. Cases in the UKCCS were children aged 0–14 years inclusive with a pathologically confirmed malignancy registered with a Family Health Services Authority (FHSA) after 1 April 1992 in England and Wales or a Health Board after 1 January 1991 in Scotland. Case accrual finished in December 1994 in Scotland and in England and Wales ended in December 1996 for leukaemias, December 1995 for non-Hodgkin lymphomas and December 1994 for other malignancies. Two controls were randomly selected per case from the same FHSA or Health Board, matched on sex and date of birth. Both cases and controls must have been born in the UK and have had no prior malignancies. 3838 eligible cases and 7629 controls participated in the UKCCS. For this analysis, we aimed to collect information on proximity to electrical supply equipment for one control per case.

### Data collection

Data on important sources of electricity supply near the homes and schools of participants were collected using an external sources questionnaire. This was designed in cooperation with National Grid Company and the regional electricity companies in England and Wales, and ScottishPower and Scottish Hydro-Electric in Scotland. The questionnaires (blinded as to case/control status) were completed by technicians from these companies, using home addresses supplied by the UKCCS.

External sources questionnaires were issued for homes in which we made measurements. Controls were assigned a pseudodiagnosis date: the date on which they were exactly the same age as the corresponding case at diagnosis. To be eligible for magnetic field

*Received 21 August 2000*
*Revised 27 September 2000*
*Accepted 27 September 2000*

*Correspondence to:* Professor N. Day, Strangeways Research Laboratory, University of Cambridge, Wort's Causeway, Cambridge CB1 8RN, UK.
E-mail: nick.day@srl.cam.ac.uk measurements, a child must have lived in the same home for the 12 months before diagnosis or pseudodiagnosis, and have still been living there at the time of measurement. For children whose family were interviewed and who were ineligible for, or whose family refused, magnetic field measurements, we issued a questionnaire for the home lived in the longest in the year prior to (pseudo) diagnosis. In this analysis, we examine all homes for which we have external sources information. This gives 3380 cases (88% of those interviewed) and 3390 controls, compared to 2226 case–control pairs for whom we also had measurements.

The external sources questionnaires were designed to identify high-voltage lines, underground cables, substations and distribution circuits that might be capable of producing magnetic field levels or 0.1 μT and above at the address of interest and to obtain load and other circuit information to enable reconstruction of historical exposure from these sources. Where direct measurements of magnetic field levels were made, we previously (UK Childhood Cancer Study Investigators, 1999) used some of these data to make adjustments for changes in the contributions from external sources between the year of interest and the time of measurement, if load data were available. Here, we examine the questionnaire data as variables of interest in themselves, considering both proximity and calculated magnetic fields.

Distance and circuit information were collected for 275 kV and 400 kV lines within 400 m and operating substations and phase-separated underground cables of 33 kV and above within 20 m of the homes of interest. The threshold distances for lower-voltage overhead lines were based on design ratings and circuit type. The highest distances were 80 m for 11 kV, 20 kV and 33 kV overhead lines and 200 m for 66 kV and 132 kV lines. The minimum catchment distances used were 100 m for 66 kV and 132 kV lines and 50 m for 11 kV, 20 kV and 33 kV lines. The presence or absence of three-phase low-voltage open-wire distribution circuits within 2 m of the exterior wall was noted, though the actual distance was not collected.

We requested load data from the appropriate electricity company for overhead lines and underground cables of 66 kV or more likely to produce magnetic field levels of 0.1 μT or above, based on voltage, distance and other operational information, including relative circuit phasing.

### Distance

A complete residential history was supplied by parents at UKCCS interview. For each subject, the home occupied for the longest in the year prior to (pseudo)diagnosis was identified. Details of these homes, including the full postal address and a grid reference, were provided on the questionnaires sent to the regional electricity companies. The companies then used their own maps and geographical information systems in order to check the addresses against the position of power lines. On rare occasions, company staff made site visits to examine distances. A two-stage process was used to identify the homes near to the higher-voltage National Grid Company (NGC) lines. Firstly, UKCCS staff used large scale (1:50000) maps provided by NGC to determine whether a home was located within 400 m of a line. Secondly, each regional electricity company was asked to identify any NGC lines within 400 m of a home and, if necessary, to supply a detailed map (1:2500 or less) showing the position of the residence. The details of homes identified by either method as being near an NGC line were then sent to NGC staff, who made a more precise assessment of distance.

### Calculated magnetic fields

In our original analysis we included an adjustment for historical line load conditions, to improve our assessment of magnetic field exposure through contemporary short-term measurements. The adjustment also allowed for other magnetic field sources in the home such as those arising from currents in local distribution circuits and household wiring. In this study, we have calculated the magnetic fields from power lines alone. We have details of nearby electrical installations for most cases in the UKCCS and are able to calculate magnetic fields for additional homes in which we were unable to make measurements.

National Grid Company's EM2D program was used to compute the power line magnetic field levels. Most computations were two-dimensional, in which the conductors were assumed to be straight, parallel and horizontal. The factors included in the model were distance between power line and home, phase arrangement, tower design and conductor clearance. The information was extracted from the external source questionnaires that were completed by the electricity companies. Where the course of a line altered near a home, a three-dimensional version of the program was used. The calculations were performed for all the cases and controls that were identified living near to power lines. Where load data had been received previously, the magnetic flux density was estimated from the load data received for the period at home during the year of interest or the nearest year available. Otherwise, a nominal load value was assigned either to reflect the annual average load conditions found on similar voltage lines in the study, or to reflect typical operational loads. If more than one power line was close to a home, the combined effect was estimated using a root sum of squares method.

### Statistical analysis

Of the 3380 cases and 3390 controls, there were 81 unmatched cases and 71 unmatched controls. This was because residential histories were occasionally incomplete or addresses impossible to locate. Owing to this, we ignored the matching and used unconditional logistic regression to estimate the risk, adjusting for the matching variables in the form of age in years, sex and UKCCS region. We also fitted the small-area seven-level deprivation index, based on unemployment, overcrowding and car ownership used in our analysis of measured magnetic fields (UK Childhood Cancer Study Investigators, 1999). All controls were used in each analysis. The data were presented by voltage and distance. We examined the effect of distance for each voltage, independently of magnetic field exposure. Using a categorical measure of distance for every voltage would give small numbers in each category. We therefore decided to model distance as a continuous variable. We used a multiple of distance$^{-1}$ as the continuous measure, because this function models a decreasing effect with increasing distance and gives greater weight to closer observations. 100 was chosen as a scaling factor. Thus, our exposure measure was taken as ($100 \times$ distance$^{-1}$ in metres), or zero if no source was reported. For each type of source (overhead line, underground cable or substation), a separate variable was used for each voltage. If two

 *© 2000 Cancer Research Campaign*

**Table 1** Distribution of distance by source among cases and controls

| ***Source*** | **ALL (%)** | **Other leukaemas (%)** | **CNS cancers (%)** | **Other malignacies (%)** | **All cases (%)** | **Controls (%)** | **Total (%)** |
|---|---|---|---|---|---|---|---|
| Overhead lines | 57 (4.3) | 9 (3.6) | 18 (2.9) | 48 (4.1) | 132 (3.9) | 125 (3.7) | 257 (3.8) |
| Underground cables | 1 (0.1) | 0 (0.0) | 1 (0.2) | 5 (0.4) | 7 (0.2) | 7 (0.2) | 14 (0.2) |
| Substations | 30 (2.3) | 3 (1.2) | 11 (1.8) | 24 (2.0) | 68 (2.0) | 62 (1.8) | 130 (1.9) |
| Low-voltage circuits | 12 (0.9) | 1 (0.4) | 2 (0.3) | 5 (0.4) | 20 (0.6) | 17 (0.5) | 37 (0.5) |
| No sources | 1242 (93.3) | 239 (95.2) | 583 (95.0) | 1112 (93.9) | 3176 (94.0) | 3195 (94.2) | 6371 (94.1) |
| All sources | 1342 ([a]100.8) | 252 ([a]100.4) | 615 ([a]100.2) | 1194 ([a]100.8) | 3403 ([a]100.7) | 3406 ([a]100.5) | 6809 ([a]100.6) |
| Number of subjects | 1331 (100.0) | 251 (100.0) | 614 (100.0) | 1184 (100.0) | 3380 (100.0) | 3390 (100.0) | 6770 (100.0) |

[a] Sum of all sources combined is more than 100% of the sample as a single home may be near more than one source

*Overhead lines*

| | 11 kV | | | | | 20 kV | | 33 kV | | |
|---|---|---|---|---|---|---|---|---|---|---|
| | ALL | Other leuk. | CNS cancers | Other malig. | Controls | CNS cancers | Controls | ALL | Other malig. | Controls |
| 0–< 10 m | 1 | 0 | 0 | 1 | 1 | 0 | 0 | 0 | 0 | 0 |
| 10–< 20 m | 2 | 0 | 2 | 5 | 9 | 0 | 0 | 0 | 0 | 1 |
| 20–< 50 m | 9 | 2 | 7 | 9 | 27 | 0 | 0 | 0 | 1 | 6 |
| 50–< 80 m | 2 | 0 | 0 | 1 | 8 | 1 | 1 | 1 | 2 | 0 |
| 80–< 120 m | | | | | | | | | | |
| 120–< 200 m | | | | | | | | | | |
| 200–< 300 m | | | | | | | | | | |
| 300–400 m | | | | | | | | | | |
| Total | 14 | 2 | 9 | 16 | 45 | 1 | 1 | 1 | 3 | 7 |
| Av. dist. (m) | 28.8 | 39.5 | 29.3 | 25.6 | 31.3 | 50.0 | 50.0 | 50.0 | 56.3 | 31.4 |

| | 66 kV | | | | 132 kV | | | | |
|---|---|---|---|---|---|---|---|---|---|
| | ALL | Other leuk. | Other malig. | Controls | ALL | Other leuk. | CNS cancers | Other malig. | Controls |
| 0–< 10 m | 0 | 0 | 0 | 0 | 0 | 0 | 0 | 1 | 0 |
| 10–< 20 m | 0 | 0 | 0 | 0 | 0 | 0 | 0 | 0 | 2 |
| 20–< 50 m | 0 | 0 | 2 | 0 | 3 | 1 | 0 | 2 | 4 |
| 50–< 80 m | 0 | 1 | 1 | 1 | 4 | 0 | 1 | 3 | 8 |
| 80–< 120 m | 5 | 1 | 0 | 2 | 1 | 0 | 1 | 2 | 9 |
| 120–< 200 m | 1 | 0 | 0 | 0 | 3 | 0 | 0 | 3 | 1 |
| 200–< 300 m | | | | | | | | | |
| 300–400 m | | | | | | | | | |
| Total | 6 | 2 | 3 | 3 | 11 | 1 | 2 | 11 | 24 |
| Av. dist. (m) | 95.3 | 65.0 | 50.3 | 83.3 | 82.8 | 37.0 | 70.5 | 87.9 | 64.1 |

| | 275 kV | | | | | 400 kV | | | | |
|---|---|---|---|---|---|---|---|---|---|---|
| | ALL | Other leuk. | CNS cancers | Other malig. | Controls | ALL | Other leuk. | CNS cancers | Other malig. | Controls |
| 0–< 10 m | 0 | 0 | 0 | 1 | 0 | 0 | 0 | 0 | 0 | 0 |
| 10–< 20 m | 0 | 0 | 0 | 0 | 0 | 0 | 0 | 0 | 0 | 0 |
| 20–< 50 m | 0 | 0 | 0 | 0 | 1 | 0 | 0 | 0 | 0 | 2 |
| 50–< 80 m | 1 | 0 | 0 | 0 | 0 | 1 | 0 | 0 | 0 | 2 |
| 80–< 120 m | 3 | 0 | 0 | 0 | 3 | 3 | 0 | 1 | 2 | 1 |
| 120–< 200 m | 1 | 0 | 0 | 2 | 7 | 1 | 0 | 0 | 2 | 3 |
| 200–< 300 m | 4 | 0 | 3 | 1 | 8 | 3 | 1 | 1 | 2 | 5 |
| 300–400 m | 1 | 2 | 0 | 0 | 4 | 7 | 1 | 1 | 5 | 9 |
| Total | 10 | 2 | 3 | 4 | 23 | 15 | 2 | 3 | 11 | 22 |
| Av. dist. (m) | 175.1 | 354.0 | 250.7 | 150.5 | 200.3 | 240.5 | 313.5 | 241.3 | 264.5 | 237.5 |

| | *Underground Cables* 33 kV | | 132 kV | | 275 kV | | | *Substations* 6.6 kV | | 11 kV | | | | | 20 kV | 33 kV | 66 kV |
|---|---|---|---|---|---|---|---|---|---|---|---|---|---|---|---|---|---|
| | Other malig. | Controls | Other malig. | Controls | ALL | CNS cancers | Controls | CNS cancers | Controls | ALL | Other leuk. | CNS cancers | Other malig. | Controls | Controls | Controls | Other malig. |
| 0–<10 m | 1 | 2 | 0 | 1 | 0 | 0 | 0 | 0 | 0 | 2 | 0 | 2 | 3 | 6 | 0 | 0 | 0 |
| 10–20 m | 3 | 2 | 1 | 1 | 1 | 1 | 1 | 1 | 2 | 27 | 3 | 8 | 18 | 50 | 1 | 1 | 1 |
| Missing | 0 | 0 | 0 | 0 | 0 | 0 | 0 | 0 | 0 | 1 | 0 | 0 | 2 | 2 | 0 | 0 | 0 |
| Total | 4 | 4 | 1 | 2 | 1 | 1 | 1 | 1 | 2 | 30 | 3 | 10 | 23 | 58 | 1 | 1 | 1 |
| Av: Dist. (m) | 13.0 | 9.75 | 13.0 | 11.5 | 18.0 | 20.0 | 10.0 | 20.0 | 14.0 | 16.2 | 15.3 | 14.2 | 15.8 | 15.3 | 18.0 | 20.0 | 20.0 |

© 2000 Cancer Research Campaign

**Table 2** Odds ratios for ALL, all leukaemia, CNS cancers, other malignancies and all malignancies for unit increase in 100 × distance$^{-1}$ from external sources by voltage, adjusted for age in years, sex, UKCCS region and deprivation index

| | Acute lymphoblastic leukaemia | | | Total leukaemia | | | Controls |
|---|---|---|---|---|---|---|---|
| | *n* | OR | 95% CI | *n* | OR | 95% CI | *n* |
| Near no equipment[a] | 1242 | 1.00 | | 1481 | 1.00 | | 3195 |
| 11 and 20 kV Lines | 13 | 0.99 | 0.89–1.10 | 15 | 0.98 | 0.88–1.08 | 46 |
| 33 kV Lines | 1 | 0.61 | 0.26–1.43 | 1 | 0.59 | 0.25–1.40 | 7 |
| 66 kV Lines | 4 | 2.76 | 0.76–10.0 | 5 | 3.15 | 1.02–9.68 | 3 |
| 132 kV Lines | 10 | 0.98 | 0.72–1.35 | 11 | 0.97 | 0.72–1.32 | 23 |
| 275 kV Lines | 9 | 1.13 | 0.48–2.66 | 11 | 1.06 | 0.46–2.48 | 23 |
| 400 kV Lines | 15 | 1.47 | 0.71–3.06 | 17 | 1.34 | 0.65–2.76 | 22 |
| 33 kV Cables | 0 | | | 0 | | | 4 |
| 132 kV Cables | 0 | | | 0 | | | 2 |
| 275 kV Cables | 1 | 1.00 | 0.70–1.44 | 1 | 0.99 | 0.70–1.42 | 1 |
| Substations | 29 | 1.02 | 0.97–1.07 | 32 | 1.01 | 0.96–1.05 | 60 |
| Low-voltage circuits | 12 | 1.66 | 0.78–3.54 | 13 | 1.57 | 0.75–3.27 | 17 |
| No. Subjects[b] | 1331 | | | 1582 | | | 3390 |

| | Central nervous system cancers | | | Other malignancies | | | Controls |
|---|---|---|---|---|---|---|---|
| | *n* | OR | 95% CI | *n* | OR | 95% CI | *n* |
| Near no equipment[a] | 583 | 1.00 | | 1112 | 1.00 | | 3195 |
| 11 and 20 kV Lines | 10 | 1.02 | 0.90–1.17 | 16 | 1.02 | 0.93–1.12 | 46 |
| 33 kV Lines | 0 | | | 3 | 0.93 | 0.57–1.52 | 7 |
| 66 kV Lines | 0 | | | 3 | 2.64 | 0.92–7.59 | 3 |
| 132 kV Lines | 2 | 0.67 | 0.31–1.48 | 9 | 1.09 | 0.93–1.28 | 23 |
| 275 kV Lines | 3 | 0.38 | 0.04–3.99 | 4 | 1.12 | 0.90–1.38 | 23 |
| 400 kV Lines | 3 | 0.68 | 0.15–3.16 | 11 | 0.82 | 0.33–2.05 | 22 |
| 33 kV Cables | 0 | | | 4 | 1.08 | 0.95–1.24 | 4 |
| 132 kV Cables | 0 | | | 1 | 0.97 | 0.78–1.21 | 2 |
| 275 kV Cables | 1 | 1.13 | 0.78–1.64 | 0 | | | 1 |
| Substations | 11 | 1.03 | 0.98–1.08 | 22 | 0.99 | 0.94–1.05 | 60 |
| Low Voltage Circuits | 2 | 0.74 | 0.16–3.28 | 5 | 0.90 | 0.33–2.48 | 17 |
| No. Subjects[b] | 614 | | | 1184 | | | 3390 |

| | All malignancies | | | Controls |
|---|---|---|---|---|
| | *n* | OR | 95% CI | *n* |
| Near no equipment[a] | 3176 | 1.00 | | 3195 |
| 11 and 20 kV Lines | 41 | 1.01 | 0.94–1.08 | 46 |
| 33 kV Lines | 4 | 0.70 | 0.43–1.15 | 7 |
| 66 kV Lines | 8 | 2.27 | 0.87–5.91 | 3 |
| 132 kV Lines | 22 | 1.03 | 0.89–1.19 | 23 |
| 275 kV Lines | 18 | 1.08 | 0.87–1.34 | 23 |
| 400 kV Lines | 31 | 1.05 | 0.54–2.01 | 22 |
| 33 kV Cables | 4 | 0.98 | 0.86–1.12 | 4 |
| 132 kV Cables | 1 | 0.91 | 0.72–1.15 | 2 |
| 275 kV Cables | 2 | 1.00 | 0.73–1.37 | 1 |
| Substations | 65 | 1.01 | 0.97–1.04 | 60 |
| Low Voltage Circuits | 20 | 1.21 | 0.63–2.32 | 17 |
| No. Subjects[b] | 3380 | | | 3390 |

[a]Reference category. [b]This is not necessarily equal to the total of the rows above, as a single home may be near more than one type of source.

sources of exactly the same voltage and type were reported, the distance of the nearer was used.

The odds ratios for (100 × distance$^{-1}$ in metres) are reported for a unit increase. This is equivalent to a change from d to 100d × (100+d)$^{-1}$ metres; for example from 25 to 20 m, 40 to 28.6 m or 200 to 66.7 m.

We also examined the effect of calculated magnetic fields. The categories for this investigation (< 0.1 μT, 0.1–< 0.2 μT, ≥ 0.2 μT) were selected beforehand to be the same as those we used for the measured analysis, which were based on previously reported results (UK Childhood Cancer Study Investigators, 2000). In a secondary analysis, the highest category was subdivided into 0.2–< 0.4 μT and ≥ 0.4 μT.

### RESULTS

Most individuals (*n* = 6371, 3176 cases, 3195 controls) lived in homes which were not located near to electricity supply equipment (Table 1). There were 30 households (17 cases, 13 controls) with exposures from two or more sources. These appear more than once in the table. Between them, they had 69 sources in total, giving an extra 39 records in the table. There are in fact 6770 unique households (3380 cases, 3390 controls) in this analysis. 257 overhead lines were reported, only 26 (13 cases, 13 controls) of which were within 20 m of the homes of interest, and 76 (36 cases and 40 controls) between 20 and 50 m. Few high-voltage

 *© 2000 Cancer Research Campaign*

**Table 3** Odds ratios for ALL, total leukaemia, central nervous system cancers, other malignancies and all malignancies by calculated magnetic field exposure from overhead power lines, adjusted for age in years, sex, UKCCS region and deprivation index

| | Acute lymphoblastic leukaemia | | | Total leukaemia | | | Controls |
|---|---|---|---|---|---|---|---|
| | *n* | OR | 95% CI | *n* | OR | 95% CI | *n* |
| < 0.1 μT[a] | 1323 | 1.00 | | 1574 | 1.00 | | 3371 |
| 0.1–< 0.2 μT | 6 | 1.70 | 0.58–4.97 | 6 | 1.53 | 0.52–4.46 | 8 |
| ≥ 0.2 μT | 2 | 0.51 | 0.11–2.33 | 2 | 0.41 | 0.09–1.87 | 11 |
| 0.2–<0.4 μT | 1 | 1.02 | 0.10–9.96 | 1 | 0.83 | 0.09–8.07 | 3 |
| ≥0.4 μT | 1 | 0.33 | 0.04–2.72 | 1 | 0.27 | 0.03–2.18 | 8 |
| **Total** | 1331 | | | 1582 | | | 3390 |
| | **Central nervous system cancers** | | | **Other malignancies** | | | **Controls** |
| | *n* | OR | 95% CI | *n* | OR | 95% CI | *n* |
| < 0.1 μT[+] | 613 | 1.00 | | 1177 | 1.00 | | 3371 |
| 0.1–< 0.2 μT | 0 | | | 3 | 1.10 | 0.29–4.22 | 8 |
| ≥ 0.2 μT | 1 | 0.48 | 0.06–3.76 | 4 | 0.94 | 0.29–2.99 | 11 |
| 0.2–< 0.4 μT | 1 | 1.79 | 0.18–17.8 | 1 | 0.76 | 0.08–7.38 | 3 |
| ≥ 0.4 μT | 0 | | | 3 | 1.01 | 0.26–3.91 | 8 |
| **Total** | 614 | | | 1184 | | | 3390 |
| | **Total malignancies** | | | **Controls** | | | |
| | *n* | OR | 95% CI | *n* | | | |
| < 0.1 μT[a] | 3364 | 1.00 | | 3371 | | | |
| 0.1–< 0.2 μT | 9 | 1.18 | 0.45–3.06 | 8 | | | |
| ≥ 0.2 μT | 7 | 0.62 | 0.24–1.61 | 11 | | | |
| 0.2–< 0.4 μT | 3 | 0.99 | 0.20–4.92 | 3 | | | |
| ≥0.4 μT | 4 | 0.49 | 0.15–1.63 | 8 | | | |
| Total | 3380 | | | 3390 | | | |

[a] Reference categor.y phase-separated underground cables within 20 m were reported (*n* = 14, seven cases and seven controls). 130 substations were reported (68 near a case home, 62 a control), 13 of which (seven cases, six controls) were within 10 m of a home of interest. No distance was supplied for five of the substations (three cases, two controls) and these were not used in the analysis. 20 cases and 17 controls had a low-voltage open-wire distribution circuit within 2 m of the exterior wall. There were 14 mixed dual-circuit lines (400/275 kV), which were included in the 400 kV line category, 10 near a case home and four near a control home. Overall, the distributions of distances were similar for cases and controls. This is confirmed by the average distances also presented in Table 1.

Examining distance alone, a separate value of 100 × distance$^{-1}$ in metres as a continuous variable (0 if no exposure reported) was used for each voltage within each type of source, exceptions being that 11 and 20 kV lines were combined (there were only two 20 kV lines reported) and substations of all voltages were combined into a single category (all but six were 11 kV), and presence of a low-voltage circuit, which was a binary variable. The results are presented in Table 2. As we used the distance of the nearer if two sources of exactly the same voltage and type were present, the total number of lines given in Table 2 is lower than in Table 1 because houses near more than one line of a particular voltage are listed only once. Homes near more than one line of *differing* voltages are still reported more than once; the 248 lines listed in Table 2 (124 for cases and 124 for controls) come from 234 homes (117 case homes and 117 control homes). Table 2 shows no evidence of association with risk for acute lymphoblastic leukaemia (ALL), central nervous system (CNS) cancers or total malignancy for proximity to any electrical supply equipment. For all leukaemias, an adjusted odds ratio of 3.15 (1.02–9.68) was obtained for 66 kV lines. The nominal significance level for this value is 0.046. A non-significant association with 66 kV lines was also seen for other malignancies, excluding CNS cancers and leukaemias, using the same control group. There is no such result for any other voltage, and given the number of independent odds ratios in Table 2, we believe that these findings can be attributed to chance. Odds ratios of 0.73 (0.42–1.26) for ALL, 0.75 (0.45–1.25) for all leukaemias, 1.08 (0.56–2.09) for CNS cancers and 0.92 (0.64–1.34) for all malignancies were obtained for a simple categorical analysis on the presence or absence of an overhead line within 50 m of the child's home (same covariates fitted, table not shown). 72% of the lines within 50 m (84 of 116) were lower voltage 11 kV or 20 kV lines. Repeating the analysis without these lines made little difference to the results, with odds ration of 0.69 (0.25–1.90) for ALL and 0.89 (0.44–1.80) for all malignancies.

Table 3 examines the risk associated with different levels of calculated magnetic field exposure from overhead power lines, for ALL, all leukaemia, CNS cancers, other malignancies and all malignancies. Most observations (3364 cases, 3371 controls) were in the reference group, which consisted of subjects with calculated magnetic field exposure from overhead power lines of less than 0.1 μT. Most of the reference group (3263 cases, 3273 controls) lived in homes near which no overhead lines had been reported. Of the subjects living in homes near power lines (117 cases, 117 controls), most (101 cases, 98 controls) were in the reference category, reflecting the conservative nature of the threshold distances used for collecting details of power lines. For acute lymphoblastic

© 2000 Cancer Research Campaign leukaemia, the odds ratio for the comparison between exposure of ≥ 0.2 μT vs < 0.1 μT, is 0.51 with an upper confidence bound of 2.33, after adjustment for matching variables and an index of deprivation. For all malignancies combined, the situation is similar, with slightly narrower confidence bounds due to greater numbers. There is no evidence, for any of the malignancy categories, to support the hypothesis that, in the year before diagnosis, exposure to magnetic fields above 0.2 μT associated with overhead power lines increases risk, nor is there any suggestion of increasing risk with increasing exposure.

We had magnetic field measurements for 179 of the 234 subjects living in homes near power lines. The average first-stage measured field levels in μT for the four categories (< 0.1, 0.1–< 0.2, 0.2–< 0.4, ≥ 0.4) for these 179 were (0.047, 0.228, 0.399, 0.585) and the average computed (0.024, 0.153, 0.292, 0.701), $n$ = (155, 10, 5, 9), suggesting that the method of computing magnetic fields we used produces reasonable results, allowing for background levels.

We also performed a matched analysis of calculated fields as a check, with a smaller number of subjects (3309 case–control pairs). The results were similar: for all malignancies, an adjusted odds ratio and 95% confidence limits of 1.24 (0.48–3.22) for the category 0.1–< 0.2 μT and 0.58 (0.21–1.58) for ≥ 0.2 μT. Our analysis used the home lived in for the longest time in the year before diagnosis or pseudodiagnosis. 6115 (3154 controls and 2961 cases) of the 6770 subjects lived in the same home for all of that year. Restricting the analysis to these subjects made little difference to the results.

### DISCUSSION

Our results provide no evidence that proximity to electricity supply equipment or exposure to magnetic fields associated with such equipment is associated with an increased risk for the development of childhood leukaemia or any other childhood cancer. These results complement our investigation of measured magnetic fields (UK Childhood Cancer Study Investigators, 1999). This earlier analysis contained fewer than 50% of cases and first-choice controls and hence could have been influenced by participation bias. The study presented here, however, includes the great majority of eligible households, so that the possible effect of participation bias is small.

Our analysis of measured magnetic fields contained a Table demonstrating the use we had made of historical load data to adjust exposure estimates (Table 5, UK Childhood Cancer Study Investigators, 1999), which included only those individuals living in homes for which we had detailed line-load data. This has on occasion been interpreted incorrectly as indicating the effects of proximity. This paper gives the full treatment of proximity for UKCCS subjects.

The main hypothesis we have considered relates to close (< 50 m) proximity to power lines, based on the main body of the epidemiological evidence, which was reviewed recently by Ahlbom et al (2000). However, Fews et al (1999) suggest that corona ions emitted from high-voltage overhead power lines may increase exposure to pollutant aerosols, at distances up to several hundred metres, particularly downwind of the power line. We have data on 132 kV lines for distances up to 200 m and 275 kV and 400 kV lines up to 400 m. To investigate the corona ion hypothesis, we combined 275 kV and 400 kV lines to examine the larger distance. A categorical analysis of the presence or absence of an overhead line of 275 kV or 400 kV within 400 m of the child's home gave odds ratios of 1.42 (0.85–2.37) for ALL and 1.15 (0.76–1.74) for all malignancies. We do not at present have sufficient information on the relative positions of the houses and the lines and so cannot consider the direction of the prevailing wind, a hypothesis which the UKCCS was not designed to test.

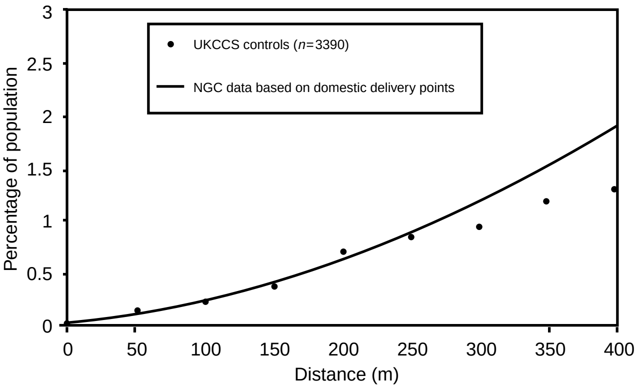

**Figure 1** Comparison of control homes reported within 400 m of transmission lines ≥ 275 kV with NGC data.

The proportions of control children living at various distances from 275–400 kV transmission lines are similar to those estimated for all UK homes by National Grid Company, at least up to 250 m (John Swanson, NGC, personal communication) (Figure 1). In our original analysis, all but one of the homes with a significant magnetic field exposure from overhead lines (those that required a load-data adjustment) were within 250 m of a transmission line. In a population-based study of adults and children in the South East of England reported by Coleman et al (1989), 0.6% of controls were within 100 m of a 132 kV or 275 kV overhead line. The corresponding proportion in our study is 0.8% (26 of 3390).

The results of studies which analysed distance alone are mixed. Coleman et al (1989) reported an increased risk of childhood and adult leukaemia with proximity to electricity supply equipment. Feychting and Ahlbom (1993) found an increased risk for leukaemia in children but for no other cancer. This association disappeared when the calculated magnetic field was included in the same model (Feychting et al, 1996), suggesting that the distance effect in the original paper could be the consequence of a magnetic field association. Tynes and Haldorsen (1997) found no increased risk for childhood leukaemia, lymphoma or brain tumours but an elevated risk for other sites. Tomenius (1986) reported an excess of visible electrical constructions within 150 m of the homes of children with cancer. Petridou et al (1997) used voltage divided by powers of distance, and found no significant elevations of risk for childhood leukaemia for higher exposure categories.

The studies examining childhood cancer and calculated fields and proximity to electrical installations, sometimes in addition to directly measured fields, have largely been European. North American research (NIEHS, 1999) has instead tended to use a wire-code approach, originally based on the work of Wertheimer and Leeper (1979; 1982). Kleinerman et al (2000) analysed the separate components used to create wire codes for the NCI study (Linet et al, 1997). They found that neither distance alone nor an exposure index based on distance and strength of multiple power

 *© 2000 Cancer Research Campaign*

lines was related to risk of ALL. Wire codes are based on a visual inspection of the type and proximity of neighbouring overhead wiring and the furthest distance reported (40 m) is thus smaller than in map-based studies of distance.

Other studies of childhood cancer and power-frequency magnetic field exposure using calculated fields (Verkasalo et al, 1993; Feychting and Ahlbom, 1993; Tynes and Haldorsen, 1997) have taken as their subjects children living in areas around power lines, where a higher-than-average proportion of homes had elevated magnetic field exposure, making it difficult to compare exposure levels directly. Olsen et al (1993) in a population-based case–control study covering the whole of Denmark, reported that 0.2% of 4788 control children had a calculated magnetic field exposure greater than 0.25 μT, which is similar to the proportion we observe (0.3%).

The statistical power of these distance and calculated field studies, including this one, has been limited; studies investigating subjects living in the vicinity of overhead lines tend to have relatively few cases and population-based studies have a small number of highly exposed individuals. We have only a few subjects living close to 132 kV or higher voltage lines, less than 120 m say, so that our study has little power to detect small effects associated with close proximity. Individually, the results of previous calculated-field studies have not been clear-cut, some finding no association, others a weakly positive one. In a recent pooled analysis, however, which examined the relationship between exposure to 50–60 Hz magnetic fields and childhood leukaemia, Ahlbom et al (2000) found a relative risk of 2.0 at exposure levels of 0.4 μT or more. This finding was based on individual records from nine studies with magnetic field measurements or calculated magnetic fields. The authors were unable, however, to exclude selection bias as an explanation for at least part of this excess. Our results are consistent with our previously reported analysis of measured magnetic fields (UK Childhood Cancer Study Investigators, 1999) in demonstrating that the great majority of exposures in the UK are at levels for which there is no evidence from the pooled data for an excess risk. We find no evidence that proximity to electrical installations or the magnetic field levels they produce in the UK is associated with increased risk of childhood leukaemia or any other cancer.

### ACKNOWLEDGEMENTS

The United Kingdom Childhood Cancer Study is sponsored and administered by the United Kingdom Coordinating Committee on Cancer Research. The Study is conducted by twelve teams of investigators (ten clinical and epidemiological and two biological) based in university departments, research institutes and the National Health Service in Scotland. The work is coordinated by a Management Committee and in Scotland by a Steering Group. It is supported by the UK Children's Cancer Study Group of paediatric oncologists and by the National Radiological Protection Board. Financial support has been provided by the Cancer Research Campaign, the Imperial Cancer Research Fund, the Leukaemia Research Fund, and the Medical Research Council through grants to their units; by the Leukaemia Research Fund, the Department of Health, the Electricity Association, the Irish Electricity Supply Board (ESB), the National Grid Company plc, and Westlakes Research (Trading) Ltd through grants for the general expenses of the study; by the Kay Kendall Leukaemia Fund for the associated laboratories studies and by the Foundation for Children with Leukaemia for the study of electric fields. The investigation in Scotland is funded principally by The Scottish Office, and by ScottishPower plc, Scottish Hydro-Electric plc and Scottish Nuclear Ltd.

We should like to thank the members of the UK Childhood Cancer Study Group for their unstinting support and the staff of local hospitals, general practitioners and general practice staff, UKCCS interviewers and technicians, members of the electricity industry in England, Wales and Scotland and staff at schools for their invaluable help. We should especially like to thank the families of the children included in the study, without whom this investigation would not have been possible.

### REFERENCES

Ahlbom A, Day N, Feychting M, Roman E, Skinner J, Dockerty J, Linet M, McBride M, Michaelis J, Olsen JH, Tynes T and Verkasalo PK (2000) A pooled analysis of magnetic fields and childhood leukaemia. *Br J Cancer* **83**(5): 692–698

Coleman MP, Bell CMJ, Taylor H-L and Primic-Zakelj M (1989) Leukaemia and residence near electricity transmission equipment: a case–control study. *Br J Cancer* **60**: 793–798

Fews AP, Henshaw DL, Wilding RJ and Keitch PA (1999) Corona ions from powerlines and increased exposure to pollutant aerosols. *Int J Radiat Biol* **75**(12): 1523–1531

Feychting M and Ahlbom A (1993) Magnetic fields and cancer in children residing near Swedish high-voltage power lines. *Am J Epidemiol* **138**(7): 467–481

Feychting M, Kaune WT, Savitz DA and Ahlbom A (1996) Estimating exposure in studies of residential magnetic fields and cancer: Importance of short-term variability, time interval between diagnosis and measurement, and distance to power line. *Epidemiol* **7**(3): 220–224

Kleinerman RA, Kaune WT, Hatch EE, Wacholder S, Linet MS, Robison LL, Shelley N and Tarone RE (2000) Are children living near high-voltage power lines at increased risk of acute lymphoblastic leukemia? *Am J Epidemiol* **151**(5): 512–515

Linet MS, Hatch EE, Kleinerman RA, Robison LL, Kaune WT, Friedman DR, Severson RK, Haines CM, Hartstock CT, Niwa S, Wacholder S and Tarone RE (1997) Residential exposure to magnetic fields and acute lymphoblastic leukemia in children. *New Engl J Med* **337**(1): 1–7

NIEHS (1999) Health effects from exposure to power-line frequency electric and magnetic fields. NIH publication no. 99–4493. NIH: North Carolina

Olsen JH, Nielson A and Schulgen G (1993) Residence near high voltage facilities and risk of cancer in children. *BMJ* **307**: 891–895

Petridou E, Trichopoulos D, Kravaritis A, Pourtsidis A, Dessypris N, Skalkidis Y, Kogevinas M, Kalmanti M, Koliouskas D, Kosmidis H, Panagiotou JP, Piperopoulou F, Tzortzatou F and Kalapothaki V (1997) Electrical power lines and childhood leukemia: a study from Greece. *Int J Cancer* **63**: 345–348

Tomenius L (1986) 50-Hz electromagnetic environment and the incidence of childhood tumors in Stockholm county. *Bioelectromagnetics* **7**: 191–207

Tynes T and Haldorsen T (1997) Electromagnetic fields and cancer in children residing near Norwegian high-voltage power lines. *Am J Epidemiol* **145**(3): 219–226

UK Childhood Cancer Study Investigators (1999) Exposure to power-frequency magnetic fields and the risk of childhood cancer. *Lancet* **354**: 1925–1931

UK Childhood Cancer Study Investigators (2000) The United Kingdom Childhood Cancer Study: objectives, materials and methods. *Br J Cancer* **82**(5): 1073–1102

Verkasalo PK, Pukkala E, Hongisto MY, Valjus JE, Järvinen PJ, Heikkilä KV and Koskenvuo M (1993) Risk of cancer in Finnish children living close to power lines. *BMJ* **307**(9): 895–899

Wertheimer N and Leeper E (1979) Electrical wiring configurations and childhood cancer. *Am J Epidemiol* **109**(3): 273–283

Wertheimer N and Leeper E (1982) Adult cancer related to electrical wires near the home. *Int J Epidemiol* **11**: 345–355

### APPENDIX

Management Committee KK Cheng, Central region; NE Day, East Anglia region; R Cartwright and A Craft, North East region;

JM Birch and OB Eden, North West region; PA McKinney, Scotland; J Peto, South East region; V Beral and E Roman, South Midlands region; P Elwood, South Wales region; FE Alexander, South West region; CED Chilvers, Trent region; R Doll, Epidemiological Studies Unit, University of Oxford, Oxford; CM Taylor, immunogenetics Laboratory, University of Manchester, Manchester; M Greaves, Leukaemia Research Fund Centre, Institute of Cancer Research; D Goodhead, Radiation and Genome Stability Unit, Medical Research Council, Harwell; FA Fry, National Radiological Protection Board; G Adams, UK Coordinating Committee for Cancer Research

Regional Investigators KK Cheng and E Gilman Central region; NE Day J Skinner and D Williams, East Anglia region; R Cartwright and A Craft, North East region; JM Birch and OB Eden, North West region; PA McKinney, Scotland; J Deacon and J Peto, South East region; V Beral and E Roman, South Midlands region; P Elwood, South Wales region; FE Alexander and M Mott, South West region; CED Chilvers and K Muir, Trent region

Leukaemia Research Fund Data Management Processing Group R Cartwright, G Law, J Simpson and E Roman.

*© 2000 Cancer Research Campaign*
